# Preliminary Studies on the Predation of the Mite *Blattisocius mali* (Acari: Blattisociidae) on Various Life Stages of Spider Mite, Thrips and Fruit Fly

**DOI:** 10.3390/insects14090747

**Published:** 2023-09-06

**Authors:** Katarzyna Michalska, Manoj Kumar Jena, Agnieszka Mrowińska, Piotr Nowakowski, Daria Maciejewska, Klaudia Ziółkowska, Marcin Studnicki, Marcin Wit

**Affiliations:** 1 Department of Plant Protection, Institute of Horticultural Sciences, Warsaw University of Life Sciences, Nowoursynowska 159, 02-776 Warsaw, Poland; jenamanoj401@gmail.com (M.K.J.); piotr.nowakowski0996@gmail.com (P.N.); daria_maciejewska@sggw.edu.pl (D.M.); marcin_wit@sggw.edu.pl (M.W.); 2 Department of Biometry, Institute of Agriculture, Warsaw University of Life Sciences, Nowoursynowska 159, 02-776 Warsaw, Poland; marcin_studnicki@sggw.edu.pl

**Keywords:** *Blattisocius*, *Tetranychus urticae*, *Frankliniella occidentalis*, *Drosophila hydei*, predatory mite, feeding rate, predation

## Abstract

**Simple Summary:**

Although our knowledge of the interactions between the underground and aboveground communities of organisms is growing, little is known about the impact of soil predatory mites on aboveground communities of plants, herbivores and their natural enemies. *Blattisocius mali* is a polyphagous predatory mite that disperses on the bodies of drosophilid fruit flies, on which it feeds; after disembarkment, it also preys on their eggs. This mite is mostly associated with soil or litter, but it has also been found in fruit and seed storage sites and on plants. In our tests, the starved predatory females readily fed on various stages of the common herbivores two-spotted spider mite and western flower thrips as well as the fruit fly *Drosophila hydei*. The predator came from cultures fed on acarid mites, and its identity was validated molecularly. Although *B. mali* shows the potential to prey upon herbivorous insects and mites, to determine whether it can also effectively reduce their population densities, further studies, including tests on the predator’s survival, fecundity and prey preference, are required.

**Abstract:**

Research in recent years has shown that some species of predatory mites, considered to be typically associated with soil and litter, can also be found on plants. Such species include *Blattisocius mali*, which is an effective predator of acarid mites, nematodes and the eggs of moths and which can disperse by means of drosophilid fruit flies. Apart from soil and litter or storage, it has also been recorded on the bark of apple trees and the leaves of strawberries, thus suggesting its possible predation of/feeding on herbivorous mites and insects. Our goal was to examine whether *B. mali* could consume different development stages of two polyphagous herbivores, the two-spotted spider mite, *Tetranychus urticae*, and the western flower thrips, *Frankliniella occidentalis*, as well as the drosophilid fruit fly *Drosophila hydei*. In 24 h cage tests, single, starved *B. mali* females consumed all types of prey offered, i.e., the eggs, males and females of spider mites; the first-instar larvae and prepupae of thrips; and the eggs and first-instar larvae of fruit flies. The potential for *B. mali* to prey upon these insects and mites was confirmed. However, to estimate whether it can also effectively reduce their population, additional tests on the predator’s survival, fecundity and prey preference are needed.

## 1. Introduction

There is a growing body of evidence concerning interactions between the underground and aboveground communities of organisms and the significant implications of these interactions for both community- and ecosystem-level properties [1,2]. With respect to this, the effect of soil predatory mites on aboveground communities including plants, herbivores and their natural enemies is still poorly understood [3].

*Blattisocius mali* (Oudemans) is an effective predator of acarid mites, nematodes and the eggs of the potato tuber moth [4,5,6,7]. It belongs to Blattisociidae, a family of predatory mites recorded in soil and on plants, associated with insects, rodents and birds. Species of the genus *Blattisocius* Keegan are a distinctive group that commonly inhabit storage facilities and feed on mites and insect eggs [8,9]. Although generally regarded as edaphic, they can visit plants; for example, *B. dendriticus* Berlese has been noted on the leaves of lychee [10], leaves/twigs/flowers of some citrus trees [11] and leaflets of strawberries [3]. So far, *B. mali* has been found in soil from agricultural fields [7,12] and in storage sites of grass seeds, potatoes and fresh and dried fruit [4,5,13,14] as well as on the bark of apple trees [15] and on strawberry leaves [16]. The presence of this predator on plants suggests that it may also prey upon herbivorous insects and mites. Interestingly, recent studies on *Drosophila melanogaster* Meigen and *D. hydei* Sturtevant have shown that *B. mali* females can not only disperse by means of drosophilid fruit flies but also feed on their bodies during transportation, and they may also prey on their eggs after disembarkment [9,17]. As predatory mites transported by insects can prey upon the juvenile stages of their carriers, mostly eggs and larvae [18,19,20], it was suspected that *B. mali* may also feed on both the eggs and larvae of drosophilids.

Our aim was to examine whether *B. mali* can prey upon the various development stages of two phytophages, the two-spotted spider mite, *Tetranychus urticae* Koch, and the western flower thrips, *Frankliniella occidentalis* (Pergande), as well as on the drosophilid fruit fly *D. hydei*. Two-spotted spider mites and western flower thrips are highly polyphagous herbivores which can also be serious pests of many crop plants [21,22,23,24]. Both species can be preyed upon by phytoseiid mites. However, as thrips prepupae and pupae hide in soil, apart from phytoseiids, some edaphic laelapid mites, e.g., *Gaeolaelaps aculeifer* (Canestrini) *and Stratiolaelaps scimitus* (Womersley), are recommended for thrips control [21,25,26]. *Drosophila hydei* is a cosmopolitan, omnivorous fruit fly commonly found in woodlands but also near or in human dwellings [27,28,29].

In this paper, we examined the 24 h feeding rate of starved *B. mali* females on the eggs, males and females of spider mites, first-instar larvae and prepupae of thrips as well as the eggs and first-instar larvae of fruit flies.

## 2. Materials and Methods

### 2.1. Biological Material and General Methods

All insects and mites were obtained from and maintained in the Section of Applied Entomology, Department of Plant Protection at Warsaw University of Life Sciences (WULS). *Blattisocius mali* was reared using a mixture of bran and various stages of the mould mite *Tyrophagus putrescentiae* (Schrank) fed on yeast, following Michalska et al. [9,17]. The species of predatory mite was identified morphologically by Prof. D.J. Gwiazdowicz and then confirmed molecularly by sequencing the cytochrome c oxidase subunit I gene fragment (COI, DNA barcode) using a method previously described by Dabert et al. [30]. 

The western flower thrip, *F. occidentalis*, was reared on cucumber plants cv. ‘Skierniewicki’ in a glasshouse, while the two-spotted spider mite, *T. urticae,* was reared on plants of the common bean cv. ‘Ferrari’ in a growth chamber at 23 °C, 70–75% RH and 16/8 h (L/D) photoperiod.

As in previous studies by Michalska et al. [9,17], we used the flightless form of the fruit fly *D. hydei*, commercially distributed as a live pet food. The population of *D. hydei* was maintained on the standard fruit fly medium based on cornmeal, molasses, yeast and propionic acid in an incubator at 25 °C with a 12/12 (L/D) photoperiod.

**Figure 1:**
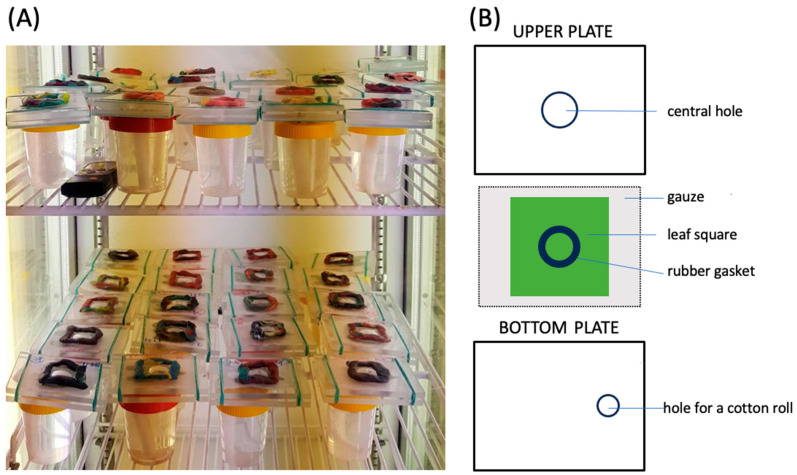
Plexiglass detached leaf cages used in the experiments. (**A**) Cages put onto containers filled with water and moistened by means of dental cotton rolls. (**B**) Plan view of detached leaf cage elements. For details, see Materials and Methods section.

For the experiments, we used glass cages with conical chambers of 0.8 cm and 0.3 cm diameter [9,17] or plexiglass detached leaf cages similar to those described by Tashiro [31] and Michalska and Studnicki [32] but consisting of only two plates (98 mm × 74 mm × 6 mm) with a central hole within the upper plate 20 mm in diameter and sealed from above with a square of ‘breathing’ dialysis cellulose membrane (Sigma-Aldrich, Steinheim, Germany, D-9402) (Figure 1). The membrane was attached to the upper plate by means of paraffin, and the edges of the membrane were additionally pressed against the plate with plasticine. Detached ‘clean’ bean leaves cv. ‘Ferrari’ with the main vein running down the middle or cucumber leaf (cv. ‘Skierniewicki’) squares (ca. 70 mm × 70 mm) were placed onto the lower plate of the plexiglass cages along with a piece of wet gauze with the underside face up. A rubber band gasket sealed the upper plate and the central hole, with a leaf forming a leaf chamber. The leaves were moistened by means of a dental cotton roll, which was placed in a container filled with water and which touched the gauze through a hole in the bottom plate (Figure 1). 

All experiments were carried out for 24 h within an incubator at 23 °C, 70–75% RH and 16/8 h (L/D) photoperiod using one-day-starved *B. mali* females. For starvation, females were put singly into the glass cages and kept there for the next 24 h. Cages with starving mites were kept in a desiccator, which provided a humidity of 75–80% RH [9,17]. The desiccator was maintained in an incubator at 23 °C and with a 16/8 h (L/D) photoperiod. 

All mites and insects were randomly selected and transferred using a fine brush. All manipulations and observations were made under an Olympus SZX 12 dissecting microscope fitted with an Olympus Highlight 3100 cold light source. Apart from treatment combinations, we prepared the control cages (without predator), and the same number of replications was carried out for both. All life stages of insects and mites were given to the predator in *ad libitum* numbers. The prey numbers per cage were based on the results of our previous pilot tests. After 24 h, from the moment of releasing the predator into the cage, the number of dead prey individuals were counted. On rare occasions, predators were found dead, and such replications were excluded from further analysis. Similarly, the dead prey individuals were counted in the control cages. In the cases of spider mites and fruit flies, the eggs with visible chorion collapse were considered dead. 

### 2.2. Experiments

For the test on *B. mali* feeding on spider mite eggs, we used ‘1.5-day’ eggs produced by spider mite females randomly selected from a population and placed on single detached bean leaves 36 h prior to the test in Petri dishes (9 cm diameter) lined up with wet cotton. The Petri dishes, each with 10 spider mite females, were kept in an incubator at 23 °C, 70–75% RH and 16/8 h (L/D) photoperiod. For the test, we used plexiglass cages with a detached bean leaf, and each cage had *n* = 20 spider mite eggs evenly distributed on a leaf arena. For both treatment and control combinations, N = 30 replications were made. 

For the experiment on predators feeding on spider mite adults, we used either females, *n* = 15 individuals per plexiglass cage with detached bean leaf, or males, also *n* = 15 individuals per cage. Both the treatment and control combination were repeated, N = 28 times with the spider mite females and N = 26 times with the spider mite males. As spider mite females laid eggs during the test, we also examined the number of consumed (totally deflated) eggs as well as the total number of eggs (including those deflated) laid by females within each cage. On this basis, for treatment combination, the mean total number of eggs laid (and offered) per cage and the mean number of destroyed eggs per cage were calculated.

The first-instar thrips larvae (*n* = 12 individuals per cage) and prepupae *(n* = 3 individuals per cage), were tested in plexiglass cages with a square of the detached cucumber leaf. The larvae were directly collected from the infested leaves of cucumber plants grown in glasshouses. We selected the first-instar larvae at their very early stage, when they were still whitish in colour. To obtain prepupae, we placed the larvae in plexiglass cages with cucumber leaves (5 individuals per cage) and kept them for the next 5–6 days in an incubator at 23 °C, 70–75% RH and 16/8 h (L/D) photoperiod. Both treatment and control combinations with either the first-instar thrips larvae or prepupae were replicated N = 22 times.

The test of *B. mali* feeding on fruit fly eggs was conducted using ‘8-hour’ eggs of the flightless *D. hydei* and following the procedure developed by Michalska et al. [9]. The eggs were obtained from random fruit fly females released into Petri dishes half-filled with 0.75% agar medium with the addition of grape juice and yeast [33]. For egg laying, the Petri dishes with flies were kept in an incubator in darkness at 25 °C for 8 h. For the test, the eggs were first rinsed with a drop of distilled water (to remove agar residues) and then placed in glass cages (*n* = 40 eggs/cage). For both treatment and control combination, N = 16 replications were carried out.

In the pilot test, we estimated that the duration of the first-instar larvae of the flightless fruit fly *D. hydei* was around 48 h. Therefore, the test of *B. mali* feeding on first-instar fruit fly larvae was undertaken using ‘12-h’ larvae of the flightless *D. hydei* so that the larvae remained at the first-instar stage until the end of the test. Our experiment was conducted following the ‘spoon method’ developed by [34], with slight modifications. The spoon was previously filled with 0.75% agar medium with the addition of grape juice and yeast [33]. For egg laying, each spoon was covered with fresh yeast and kept along with 9- to 18-day-old flies for 20–24 h in a half-pint bottle in an incubator with a 12/12 (L/D) photoperiod at 25 °C. Then, the spoons were removed from the bottles and kept in the incubator, in the same conditions as previously, for 32–34 h. Each spoon was removed and the larvae were washed into a Petri dish with distilled water. For the test, the ‘12-h’ larvae were placed in glass cages, *n* = 20 larvae per cage. Both the treatment and control combination were replicated N = 16 times.

To protect the fruit fly eggs and larvae from drying out during the tests, the filter paper at the bottom of each glass cage was heavily moistened and additionally sealed with Scotch tape to maintain humidity inside the cage. 

Statistical analysis was performed using R 4.2.1 software [35]. In order to compare the number of dead *T. urticae* males and dead *F. occidentalis* larvae or prepupae in combination with a *B. mali* female or without a predator, the one-factor generalized linear model (GLM) was applied, which enabled us to analyse the count data without their prior transformation. Due to frequent ‘0’ records of dead individuals in the control combination, the model was based on a zero infinite Poisson distribution [36]. When there was no destroyed prey in the control (only ‘zero’ records), such as in the case of drosophilid eggs and larvae as well as eggs and females of *T. urticae*, statistical analysis was not performed. The data are given as mean ± SE and the accepted level of significance is *p* < 0.05. 

## 3. Results and Discussion

Among the five barcoded predatory mite specimens, we found only one COI haplotype (GenBank acc. no. OQ825956), which differed by 2.7% (SD 0.7) from *B. mali* sequences found in specimens collected in Israel (MW344275–MW344284). Resolving whether this genetic distance can indicate a cryptic species is impossible without testing more *Blattisocius* species to determine the barcoding gap for the COI marker in the genus and confirming the results with nuclear DNA data. However, in mites, the barcoding gap is usually at a level of >10% (e.g., [37,38,39]), which supports the same-species hypothesis. Moreover, the only COI sequences published for other *Blattisocius* species, i.e., *B. tarsalis* (Berlese) (MK270529.1) and *B. keegani* Fox (MH120211.1), differ >34% between all three species in the analysed fragment.

**Figure 2:**
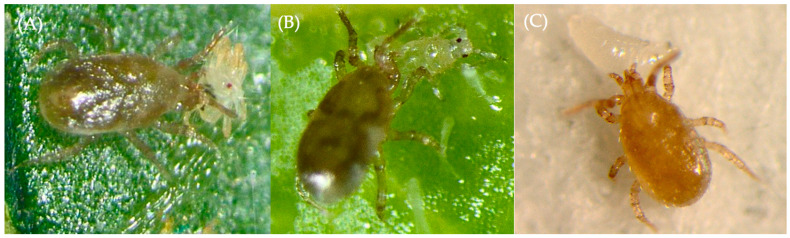
A female of *Blattisocius mali* feeding on (**A**) a male of the two-spotted spider mite, *Tetranychus urticae*, (**B**) a first-instar larva of the western flower thrips, *Frankliniella occidentalis*, and (**C**) a first-instar larva of the fruit fly *Drosopila hydei*.

This is the first report on predation of the species from the *Blattisocius* genera on herbivorous mites and insects (Figure 2). Although frequently recorded in soil, litter and fruit or vegetable storage sites, *B. mali* was first found on and described from the bark of the apple tree [4,5,7,12,14]. Moreover, it has also been collected from leaves of strawberries infested by two phytophagous mites, i.e., *T. urticae* and *Phytonemus pallidus* Banks [16]. It has been suggested that the appearance of *B. mali* on plants might not have been accidental but connected with the predator’s foraging. The two herbivores may be part of its diet, but this aspect was not investigated by the authors. The daily (mostly nocturnal) movement of the edaphic predatory mites *B. dendriticus* and *Proctolaelaps pygmaeus* (Muller) from organic mulch to strawberry plants was detected by Esteca et al. [3]. According to the authors, *B. dendriticus*, previously known to feed on acarid mites, might have been attracted by the presence of *Ty. Neiswanderi* Johnston and Bruce on strawberry leaves. However, *T. urticae* cohabited these plants. Thus, one cannot exclude that, as in the case of *B. mali*, spider mites could also be prey for this predatory mite. The probable association of *B. dendriticus* with another herbivorous mite, the lychee erinose mite, *Aceria litchii* (Keifer), which forms erinea on lychee leaves, was reported by Waite and Gerson [10]. However, its feeding on this eriophyid has not been confirmed.

The feeding rates of *B. mali* on *T. urticae* eggs, males and females are presented in Table 1. These rates appear close to those found for some generalist phytoseiid mites. For example, 24 h starved, random females of *Amblyseius swirski* Athias-Henriot and *Neoseiulus californicus* (McGregor) (obtained from the commercial Spical and Swirski-Mite Koppert biopreparates and mass-reared on *T. urticae*) exposed to 30 eggs aged 0–24 h of *T. urticae* consumed on average 14.83 and 8.84 eggs, respectively, within 24 h at ca. 25 °C [40]. In another study at 25 °C, newly mated, gravid females of the Japanese strain of *N. californicus* consumed on average 12.83 eggs (age unspecified), 6.4 males and 2.43 females of *T. urticae* per day (out of 20 prey individuals offered each day) in 7 consecutive days [41]. In our study, single, starved and random *B. mali* females, which had no previous experience with tetranychids, consumed on average 10.93 out of 20 spider mite eggs offered (0–36 h old), and when exposed to 15 spider mite females, they ate 1.96 females on average, plus 0.82 out of 68.12 eggs oviposited on average per cage (Table 1). We also noted on average 2.81 spider mite males destroyed per cage. However, the mean number of males consumed by the predator was probably slightly lower, considering that some males in the control also died during the test, though in much lower numbers than in treatment cages (GLM: χ^2^ = 35.098, df = 1, *p* < 0.0001). It should be emphasized that in the test with spider mite eggs, we did not examine the effect of the presence of webbing on the foraging efficiency of *B. mali*. Under natural conditions, spider mite eggs are often hidden under the web and laid in it, especially in the presence of predators [42]. Among phytoseiids, there are species that can move easily among dense strands of the web, while others can cope with it to a lesser extent or are even hindered by it and are usually considered ineffective predators of *Tetranychus* species [43,44]. In our tests with spider mite females, the web was clearly seen, and we noted a few cases of *B. mali* females that got stuck and died in the web. Thus, the extent to which webbing affects *B. mali* foraging and predation on *T. urticae* should be evaluated in further research. 

For the biological control of *F. occidentalis* against the aboveground larval stages of thrips, several generalist phytoseiids are recommended, including *Amblydromalus limonicus* (Garman and McGregor), *A. swirski* and *Neoseiulus cucumeris* (Oudemans) [45]. Van Houten et al. [46] tested seven phytoseiid species previously fed on pollen. At 25 °C on cucumber leaf discs with 12 *F. occidentalis* at their first larval stage, the mean feeding rates ranged from 0.5 to 6.9 thrips larvae/day, with the highest rates of 6.9 larvae for *A. limonicus*, 6.0 larvae for *N. cucumeris* and 4.4 larvae for *Iphiseius degenerans* Berlese. In another study [47], *A. swirski* was mass-reared on a mixture of pollen and spider mite eggs, and when exposed to 15 first-instar larvae of *F. occidentalis* at 25–27 °C, they consumed even more thrips on average, i.e., 9.9 larvae per day. In our study, the predation rate on thrips larvae appears to be lower than for the recommended phytoseiids (Table 1). The mean number of first-instar larvae destroyed in treatment cages was 4.73. However, the predation rate of hungry *B. mali* might have been slightly lower, considering that we also detected some mortality of thrips in control cages (GLM: χ^2^ = 43.816, df = 1, *p* < 0.0001) (Table 1). 

Our observations and the test also showed that *B. mali* females can attack and consume the prepupae of *F. occidentalis*. However, we offered the predatory females only three prepupae per cage and also recorded quite high thrips mortality in the control, almost half as high as that recorded in the treatment (GLM: χ^2^ = 3.9282, df = 1, *p* = 0.0475) (Table 1). Thus, the potential feeding rates obtained in the test might have been underestimated, as there may have also been dead prepupae in some cages, which the predator may have fed on instead of live individuals. For comparison, when offered 5 pupae of *F. occidentalis* at ca 25 °C, starved *S. scimitus* females consumed on average 1.38 pupae during 24 h [48]. Interestingly, this edaphic mite can also prey on thrips larvae on the bottom leaves of cucumber and eggplants [49]. Although it is likely that *B. mali* could control both above- and belowground stages of this pest, further studies are needed, including experiments on its effectiveness against thrips larvae, prepupae and pupae directly in the crop. 

Previous studies have shown that *B. mali* can not only feed on drosophilids during dispersal but also prey on their eggs [9,17]. In the test with 15 ‘8-hour’ eggs of *D. hydei*, 24 h starved predatory females consumed on average 6 fly eggs in 10 h, 3 of which were only partially consumed. Interestingly, some *B. mali* females returned to the partially fed eggs after some time and only then consumed them completely [9]. In this study, *B. mali* females were given 40 eggs of this fly and, within 24 h, ate on average 17 eggs, among which 5.38 ± 0.46 eggs were only partially consumed. Moreover, we demonstrated for the first time that *B. mali* can consume fruit fly larvae. When given 20 first-instar larvae of *D. hydei,* a starved female consumed on average 10.19 larvae per day (Table 1). Most larvae were consumed totally, i.e., 9.19 ± 0.49, whereas only a few larvae, i.e., 1 ± 0.32 were fed on to a lesser extent. Voracity of the edaphic predatory mites on drosophilid development stages was also examined by Esteca [50] using *D. suzukii* Magtsumura and 2-day-old gravid females of *Macrocheles embersoni* Azevedo, Castilho & Berto, *M. muscadomesticae* (Scopoli), *P. bicklei* (Bram) and *S. scimitus* previously fed with nematodes. When exposed to 20 fly eggs or 10 first-instar *D. suzukii* larvae for 10 days, the predatory females ate at 25 °C on average 2–10 eggs or 4–7 larvae per day. In these studies, however, mites were not starved before the experiment, and far fewer fruit fly eggs or larvae were offered than in our tests, which may have been the reason for the lower feeding rate in these species compared to *B. mali*. Interestingly, they also consumed the second- and third-instar larvae and, with the exception of *M. muscadomesticae*, the pupae of *D. suzukii*. 

It should be emphasized that, in natural conditions, some drosophilid eggs and larvae may be buried in the substrate. Esteca [50] found that only *S. scimitus* was able to access *D. suzukii* eggs embedded in fruit. Thus, to assess the potential negative effect of *B. mali* on the fitness of its drosophilid host, more research is needed, taking into account the predation of this mite on all development stages of fruit flies exposed to it or hidden in the substrate. 

In summary, this study revealed the potential for *B. mali* to prey upon various stages of phytophagous insects and mites as well as drosophilid fruit flies. Females starved for 24 h, previously fed on acarid mites and unfamiliar with other prey types, readily attacked and consumed the adults and eggs of two-spotted spider mites, the first-instar larvae and prepupae of western flower thrips and both the eggs and first-instar larvae of the fruit fly *D. hydei*. The 24 h feeding rate of *B. mali* on spider mites did not differ from that estimated for some polyphagous phytoseiids. Similarly, *B. mali* daily feeding rates on eggs and young larvae of *D. hydei* were comparable to those estimated for other edaphic predatory mites consuming eggs and larvae of *D. suzukii*. Only the voracity of the predator towards the larvae or prepupae of thrips was markedly lower than that found for some phytoseiid and laelapid mites. Undoubtedly, much more research is needed to estimate whether thrips, spider mites or drosophilids could be alternative prey for this mite. Future studies should be concerned with not only *B. mali* survival, fecundity and life cycle when feeding on theses insect and mites but also the predator’s prey preference and performance when foraging in their habitat.

So far, *Blattisocius mali* has been regarded as a potential bioagent of acarid mites, nematodes and potato tuber moths [4,5,6,7]. Interestingly, when *B. mali* fed on the mould mite *T. putrescentiae,* its intrinsic rate of population increase (r) was much higher than that of *B. dendriticus* or *G. aculeifer*, which are both recommended for the control of this acarid mite [4,51,52]. Thus, *B. mali* may be a promising alternative to *G. aculeifer* in the protection of bulbs of ornamental plants or cucumber plant seedlings against acarid mites [52,53], where it could additionally feed on spider mites or thrips. One also cannot exclude its potential in controlling both the adult population and offspring of drosophilid pests such as *D. suzukii* [54].

## Figures and Tables

**Table 1:** Mean number (±SE, min.–max.) of prey individuals destroyed in cages with hungry *Blattisocius mali* females after 24 h exposure to various stages of the two-spotted spider mite *Tetranychus urticae*, the western flower thrips, *Frankliniella occidentalis*, and the fruit fly *Drosophila hydei*.

Prey Type	No. of Replication N	No. of Prey Offered *n*	No. of Prey Destroyed in a Cage		Statistical Analysis for Treatment and Control Difference *
			with Predator	in Control	
*Tetranychus urticae* Eggs Males Females Eggs laid by females exposed to *B. mali* *Frankliniella occidentalis* First-instar larvae Prepupae *Drosophila hydei* Eggs First-instar larvae	30 26 28 28 22 22 16 16	20 15 15 68.12 ± 2.637 (33–92) 12 3 40 20	10.93 ± 0.58 (5–14) 2.81 ± 0.323 (1–5) 1.96 ± 0.1 (1–3) 0.82 ± 0.186 (0–3) 4.73 ± 0.39 (2–8) 0.64 ± 0.168 (0–2) 17.0 ± 0.801 (10–22) 10.19 ± 0.69 (6–13)	0 0.35 ± 0.095 (0–1) 0 0 0.23 ± 0.091 (0–1) 0.32 ± 0.102 (0–1) 0 0	- χ^2^ = 35.098, df = 1, *p* < 0.0001 - - χ^2^ = 43.816, df = 1, *p* < 0.0001 χ^2^ = 3.9282, df = 1, *p* = 0.0475 - -

* When there was no destroyed prey in control (only ‘zero’ records), the statistical analysis was not performed.

## Data Availability

The data used in this study are available by email request to the corresponding author.
